# In Vitro and In Vivo Protective Effects of Lentil (*Lens culinaris*) Extract against Oxidative Stress-Induced Hepatotoxicity

**DOI:** 10.3390/molecules27010059

**Published:** 2021-12-23

**Authors:** Yeon-Seop Jung, So-Hee Lee, So Young Chun, Dae Hwan Kim, Byung Ik Jang, Man-Hoon Han, Syng-Ook Lee

**Affiliations:** 1 Department of Food Science and Technology, Keimyung University, Daegu 42601, Korea; jys86170@dgmif.re.kr (Y.-S.J.); jy16162727@naver.com (S.-H.L.); 2 Laboratory Animal Center, Daegu-Gyeongbuk Medical Innovation Foundation, Daegu 41061, Korea; 3 BioMedical Research Institute, Kyungpook National University Hospital, Daegu 41404, Korea; soyachun99@naver.com; 4 Department of Laboratory Animal Research Support Team, Yeungnam University Medical Center, Daegu 42415, Korea; ikorando5@hanmail.net; 5 Department of Internal Medicine, Yeungnam University College of Medicine, Daegu 42415, Korea; jbi@med.yu.ac.kr; 6 Department of Pathology, School of Medicine, Kyungpook National University, Daegu 41404, Korea; one-many@hanmail.net

**Keywords:** lentil, hepatoprotective effect, oxidative stress, Nrf2

## Abstract

Excessive oxidative stress plays a role in hepatotoxicity and the pathogenesis of hepatic diseases. In our previous study, the phenolic extract of beluga lentil (BLE) showed the most potent in vitro antioxidant activity among extracts of four common varieties of lentils; thus, we hypothesized that BLE might protect liver cells against oxidative stress-induced cytotoxicity. BLE was evaluated for its protective effects against oxidative stress-induced hepatotoxicity in AML12 mouse hepatocytes and BALB/c mice. H_2_O_2_ treatment caused a marked decrease in cell viability; however, pretreatment with BLE (25–100 μg/mL) for 24 h significantly preserved the viability of H_2_O_2_-treated cells up to about 50% at 100 μg/mL. As expected, BLE dramatically reduced intracellular reactive oxygen species (ROS) levels in a dose-dependent manner in H_2_O_2_-treated cells. Further mechanistic studies demonstrated that BLE reduced cellular ROS levels, partly by increasing expression of antioxidant genes. Furthermore, pretreatment with BLE (400 mg/kg) for 2 weeks significantly reduced serum levels of alanine transaminase and triglyceride by about 49% and 40%, respectively, and increased the expression and activity of glutathione peroxidase in CCl_4_-treated BALB/c mice. These results suggest that BLE protects liver cells against oxidative stress, partly by inducing cellular antioxidant system; thus, it represents a potential source of nutraceuticals with hepatoprotective effects.

## 1. Introduction

Liver diseases have become a major global public health problem, and severe liver injury can overwhelm the self-regenerative capacity of the liver and can prove fatal [1]. Excessive oxidative stress plays a role in the pathogenesis of hepatic diseases such as alcohol-related liver disease, non-alcoholic fatty liver disease, hepatitis, fibrosis, and cirrhosis and can be induced by intrinsic factors and/or multiple xenobiotics such as alcohol and toxic compounds [1,2,3,4].

Living organisms produce reactive oxygen species (ROS) and reactive nitrogen species (RNS) via normal cellular metabolism [5]. Under normal healthy conditions, ROS and RNS generated by various pro-oxidants including alcohol [6], acetaminophen [2,4], ethyl carbamate [3], and fatty acids [7] are scavenged by various cellular enzymatic and non-enzymatic antioxidants, such as superoxide dismutase (SOD), catalase (CAT), glutathione peroxidase (GPx), glutathione reductase (GR), glutathione (GSH), and thioredoxin. These cellular antioxidants possibly play an important role in the prevention of oxidative stress-related diseases, such as liver disease [8]. However, oxidative stress occurs if the pro-oxidants exceed the antioxidant capacity of the cells and is one of the main factors associated with the development of hepatopathy through hepatocellular damage, including nucleic acid damage, protein oxidation, lipid peroxidation, and mitochondrial failure [1].

Lentils (*Lens culinaris*) have recently been gaining increasing attention as one of the top five superfoods. They are rich in protein and other essential nutrients, including folate, iron, potassium, and dietary fiber. Reportedly, lentil extracts exert multiple pharmacological activities in vitro and in vivo, such as antidiabetic, hypotensive, hypolipidemic, and cardioprotective activities [9]. A study in our laboratory has also demonstrated the direct ROS scavenging activity of phenolic extract from beluga lentil (BLE) [6]. Therefore, in this study, we hypothesized that BLE protects hepatic cells from oxidative stress-induced death. Here, we first investigated the hepatoprotective effects of BLE and the underlying mechanisms of its action in H_2_O_2_-treated AML12 mouse hepatocytes. In vivo hepatoprotective effects of BLE were further evaluated in an animal model of CCl_4_-induced acute liver failure.

## 2. Materials and Methods

### 2.1. Materials and Preparation of Lentil Extract

All chemicals were obtained from Sigma-Aldrich Chemical (St. Louis, MO, USA), unless otherwise indicated. Polyclonal antibodies to Nrf2 (sc-722) and HO-1 (#374090) were purchased from Santa Cruz Biotechnology (Santa Cruz, CA, USA) and Merck (Kenilworth, NJ, USA), respectively. Monoclonal antibody against glyceraldehyde 3-phosphate dehydrogenase (GAPDH; #2118) was purchased from Cell Signaling Technology (Danvers, MA, USA). Cell culture reagents were purchased from Gibco-BRL (Rockville, MD, USA) and Welgene (Gyeongsan, Korea). Beluga lentils were obtained from Zürsun Idaho Heirloom Beans (Twin Falls, ID, USA). Ground beluga lentil seeds (100 g) were extracted three times with 80% methanol containing 0.2% HCl (1 L) at room temperature for 24 h on an orbital shaker at 150 rpm, and the supernatants were then filtered, concentrated under reduced pressure, and lyophilized. The yield value of obtained extract was 13.25% (*w*/*w*).

### 2.2. Cell Cultures

AML12 mouse hepatocytes were purchased from the American Type Culture Collection (Rockville, MD, USA). AML12 cells were cultured in 5% CO_2_ at 37 °C in DMEM-F12 medium (Gibco-BRL) supplemented with 10% fetal bovine serum (Welgene), antibiotics, insulin-transferrin-selenium (Gibco-BRL), and dexamethasone (40 ng/mL).

### 2.3. Animals and Experimental Design

BALB/c mice (8 weeks old, male) were purchased from Orient Bio Inc. (Seoul, Korea). Animals were housed in a climate-controlled environment (21 ± 2 °C under 40–60% humidity) with a 12 h light/dark cycle and allowed to acclimatize to the facility for 7 days. The mice were then separated into the following four groups using a randomized block design method: normal control group (normal), CCl_4_ control group (CCl_4_), CCl_4_ with 100 mg/kg/day BLE (BLE100), and CCl_4_ with 400 mg/kg/day BLE (BLE400). The mice were allowed free access to food and water and a control (10 kcal% fat) pellet diet (Cat. No. D10001; Research Diets, Inc., New Brunswick, NJ, USA) was administered. The mice were treated with BLE in saline by oral gavage for 2 weeks, and food intake and body weight were measured every week. After BLE treatment for 2 weeks, all animals from each group except for the normal group received a one-time intraperitoneal injection of CCl_4_ (0.1 mL/20 g body weight, 1% in corn oil) [10]. Then, the mice were sacrificed under anesthesia 24 h after injecting CCl_4_, and their serum and tissues were aseptically removed. The serum was obtained by centrifuging at 4 °C (3000× *g* for 15 min) and then immediately subjected to biochemical analyses. For histological examination, a whole lobe of liver from each animal was fixed in 10% formalin, embedded in paraffin, processed into 4 μm-thick sections, and subjected to hematoxylin and eosin staining. The rest of the liver tissue samples were stored in a −80 °C freezer for further experiments. All animals were maintained and used in accordance with the guidelines of the Institutional Animal Care and Use Committee of the College of Medicine, Yeungnam University (YUMC-AEC2019-001).

### 2.4. Statistical Analysis

Statistical significance of differences between groups was analyzed using either Student’s *t*-test (Sigma Plot 10.0; Systat Software Inc., San Jose, CA, USA) or one-way analysis of variance with Duncan’s multiple test (statistical package for the social sciences, version 23.0, SPSS Inc., Chicago, IL, USA). The results are expressed as means with standard error of the mean (SEM; *n* ≥ 3) for each group unless otherwise indicated, and a *p* value of less than 0.05 was considered statistically significant.

**Figure 1:**
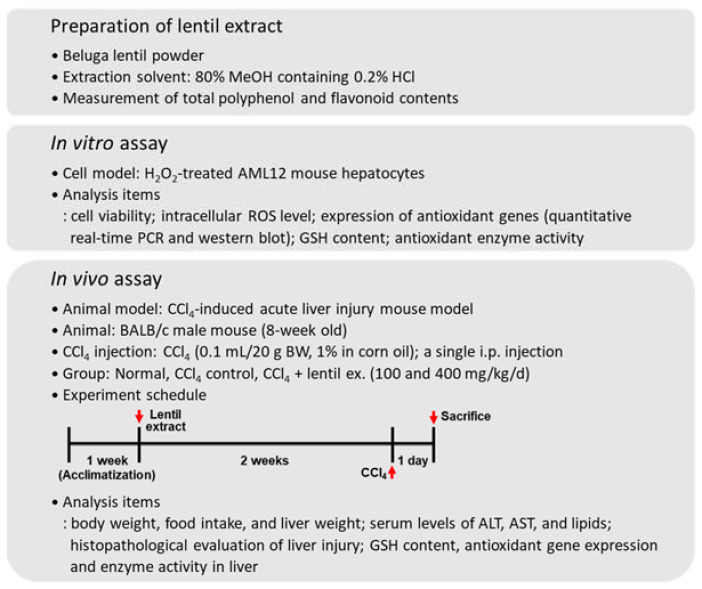
A summary of the research methodology of the study. ALT: alanine transaminase; AST: aspartate transaminase.

All other materials and methods are described in the Supplementary Materials and Methods section, and Figure 1 represents all the methodology used in this study.

## 3. Results and Discussion

### 3.1. BLE Protects AML12 Cells against Oxidative Stress-Induced Cytotoxicity

**Figure 2:**
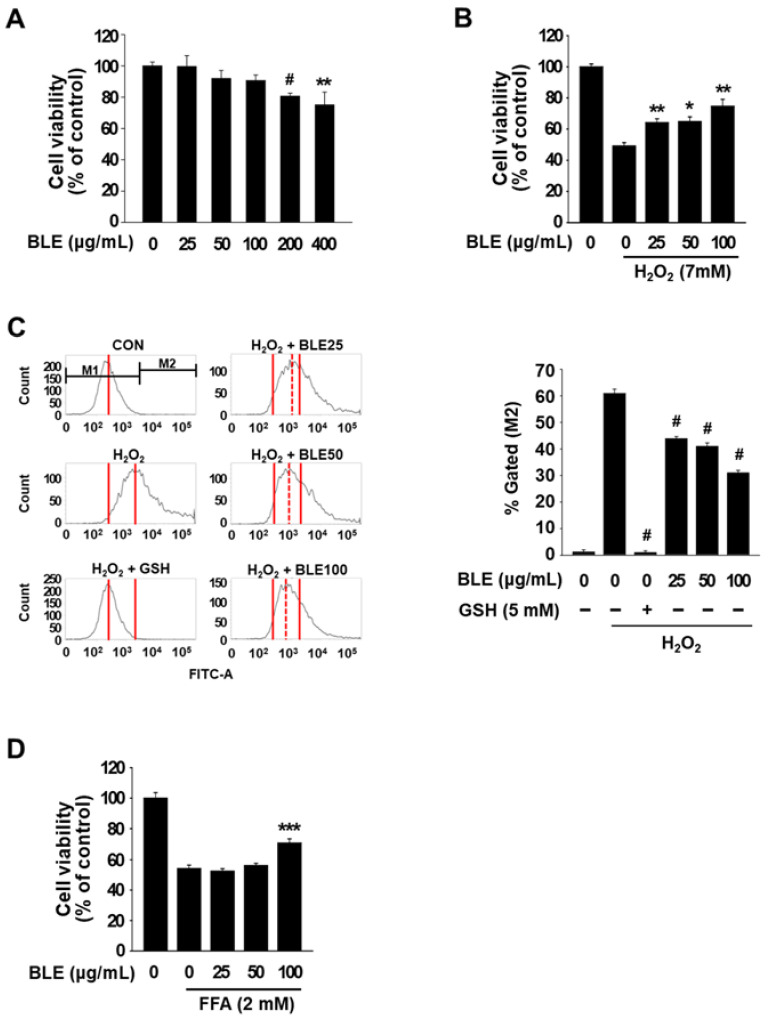
Protective effects of BLE against oxidative stress-induced cytotoxicity in AML12 cells. Cells were treated with the indicated concentrations of BLE for 24 h (**A**). After 24 h treatment with BLE, the media were replaced with new media containing H_2_O_2_ (**B**,**C**) and free fatty acids (**D**), and the cells were incubated for another 4 h. Cell viability was determined by an MTT assay (**B**,**D**) and intracellular ROS level was measured by flow cytometry (**C**). All results are expressed as means ± SEM (*n* ≥ 3). * *p* < 0.05, ** *p* < 0.01, *** *p* < 0.005, and ^#^ *p* < 0.001 vs. the H_2_O_2_ or free fatty acids (FAA) control.

BLE was prepared from ground beluga lentil seeds using 80% methanol containing 0.2% HCl, and the total polyphenol and flavonoid contents of BLE were 32.09 mg GAE/g and 19.32 mg QE/g, respectively (Table 1). Prior to investigating the protective effect of BLE on oxidative stress-induced cytotoxicity in AML12 cells, we first determined the dose-dependent cytotoxic effects of BLE (0–400 μg/mL) on AML12 cells using MTT assay. BLE at concentrations lower than 100 μg/mL had no significant cytotoxic effect on AML12 cells (Figure 2A), and thus 100 μg/mL was the highest concentration of BLE used for all subsequent experiments on AML12 cells. To examine the protective effect of BLE against oxidative stress-induced cytotoxicity, AML12 cells were pretreated with 25, 50, or 100 μg/mL of BLE for 24 h, and the media were then replaced with new media containing H_2_O_2_ (7 mM). H_2_O_2_ treatment for 4 h caused a marked decrease (about 50%) in cell viability; however, pretreatment with BLE (25–100 μg/mL) significantly preserved the viability of the H_2_O_2_-treated cells (Figure 2B). As shown in Figure 2C, the levels of intracellular ROS were significantly increased in cells treated with H_2_O_2_, compared with untreated control cells. However, pretreatment with BLE (25, 50, and 100 μg/mL) or reduced GSH (a positive control) significantly decreased cellular ROS levels in H_2_O_2_-treated AML12 cells. These data indicate that BLE protected AML12 cells from H_2_O_2_-induced cytotoxicity by reducing ROS generation.

We further investigated whether BLE can protect AML12 cells from cytotoxicity induced by free fatty acids (FFA; palmitic acid:oleic acid/1:1), which is a different type of pro-oxidant known to induce hepatotoxicity [7,11]. As shown in Figure 2D, FFA (2 mM) showed cytotoxicity in AML12 cells and pretreatment with 100 μg/mL BLE significantly protected cells from FFA-induced toxicity, whereas <50 μg/mL BLE did not show a protective effect. In a previous study, we showed that the pretreatment of AML 12 cells with 50 and 100 μg/mL BLE significantly protected the cells from alcohol-induced toxicity [6]. Previous research has also reported that extracts from various lentil cultivars protect cells from toxicity induced by different types of prooxidant, including Fenton’s reagent and antiotensin II [12,13]. These results suggest that BLE has the ability to protect hepatocytes from the cytotoxicity induced by a broad spectrum of pro-oxidants; however, there may be some differences in effective dose depending on the type of pro-oxidants. In addition, BLE may have the potential to prevent or treat liver disorders caused by different pro-oxidants, including alcoholic fatty liver, alcoholic hepatitis, and non-alcoholic fatty liver.

### 3.2. BLE Upregulates the Expression of Antioxidant Genes, in Part, via the Activation of Nrf2 in AML12 Cells

**Figure 3:**
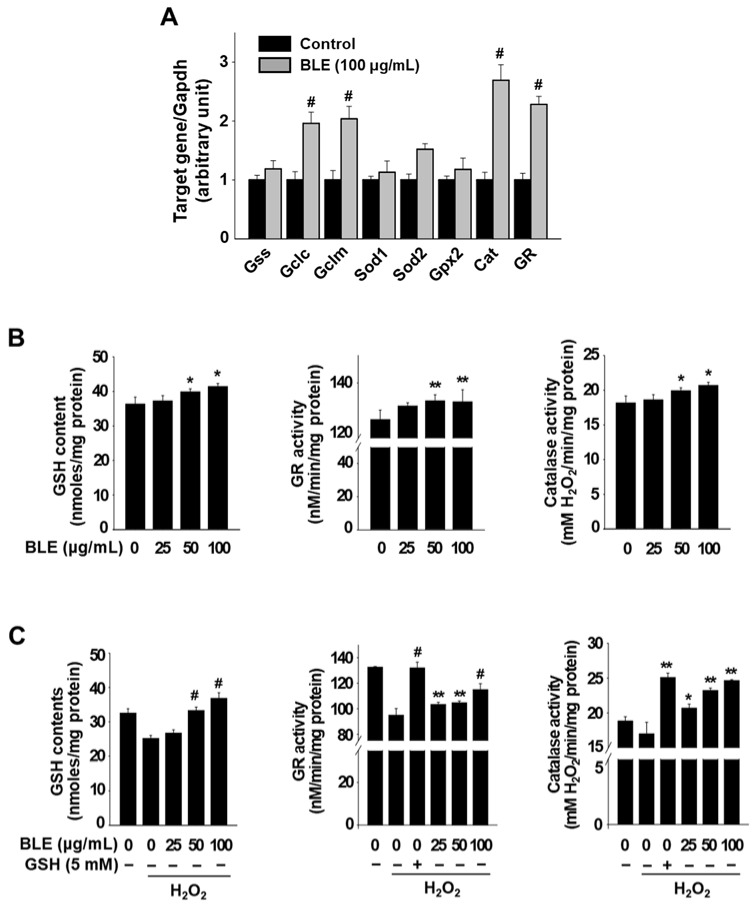
Effects of BLE on mRNA expression and activity of cellular antioxidant enzymes in AML12 cells. Cells were treated with BLE (100 μg/mL) for 18 h, and mRNA expression levels were determined by quantitative real-time PCR (**A**). Gapdh was used as an internal control. After 24 h treatment with the indicated concentrations of BLE, GSH content and enzyme activity in cells were measured (**B**). Cells were treated with the indicated concentrations of BLE for 24 h. The cells were then incubated for another hour with new media containing H_2_O_2_, and GSH contents, Gr activity, and Cat activity in cells were measured (**C**). All results are expressed as mean ± SEM (*n* ≥ 3). * *p* < 0.05, ** *p* < 0.01, and ^#^ *p* < 0.001 vs. the DMSO (**A**,**B**) or H_2_O_2_ control (**C**).

To investigate the underlying mechanism that contributes to the reduction in cellular ROS by BLE in H_2_O_2_-treated AML12 cells, we first examined the effects of BLE on the mRNA expression of eight antioxidant-related genes. We identified four genes, *Gclc*, *Gclm*, *Cat*, and *Gr*, which had >2-fold increased expression in cells treated with BLE for 18 h when compared with untreated control cells (Figure 3A). *Gclc* and *Gclm* are the genes involved in GSH synthesis, and the increased mRNA levels of these two genes was consistent with the level of total cellular GSH content in AML12 cells treated with 50 and 100 μg/mL BLE (Figure 3B). It was also confirmed that the enzyme activity of Cat and Gr was also significantly increased by BLE treatment (50 and 100 μg/mL) in AML12 cells (Figure 3B). We further examined the effect of BLE on cellular GSH content and enzyme activity of Cat and Gr in H_2_O_2_-treated AML12 cells. As shown in Figure 3C, H_2_O_2_ treatment for 1 h caused a marked decrease in GSH content; however, pretreatment with BLE (50 and 100 μg/mL), but not 25 μg/mL, significantly restored GSH content in H_2_O_2_-treated AML12 cells. The enzyme activity of Cat and Gr was also decreased in cells treated with H_2_O_2_, compared with untreated control cells. However, pretreatment with BLE (25, 50, and 100 μg/mL) or reduced GSH (a positive control) significantly increased the enzyme activity in H_2_O_2_-treated AML12 cells (Figure 3C). These results suggest that pretreatment with BLE reduced intracellular ROS levels, in part, by increasing the total GSH content and enzyme activity of CAT and GR in H_2_O_2_-treated AML12 cells.

In previous studies, lentil extracts have been shown to exhibit chemical- and cell-based antioxidant activities against multiple types of ROS [13,14,15] and have also been shown to modulate intracellular ROS levels and protect cells from ROS-induced cytotoxicity in many different primary cells and cell lines, including primary cardiomyocytes (human and rat), U373 human glioblastoma cells, and PC12 rat pheochromocytoma cells [13,14,15]. However, none of the studies revealed their cellular antioxidant mechanisms, thus, to our knowledge, this is the first report of lentil extract-mediated cellular antioxidant mechanisms in vitro.

**Figure 4:**
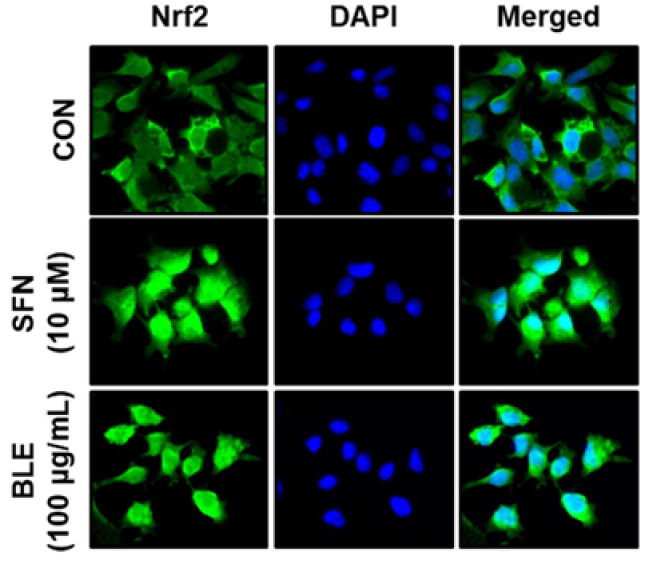
Effects of BLE on activation of Nrf2 in AML12 cells. Cells were treated with the indicated concentrations of BLE or sulforaphane (SFN) for 6 h, and endogenous Nrf2 was detected by indirect immunofluorescence staining with anti-Nrf2 antibody (×400 magnification).

Nrf2 acts as a transcriptional activator of antioxidant genes by binding to antioxidant response elements that are present in the regulatory regions of a range of antioxidation-related genes [16,17]. Thus, the effects of BLE on the expression and nuclear translocation (activation) of Nrf2 were investigated. The protein expression of Nrf2 was dramatically increased following treatment with BLE (25, 50, and 100 μg/mL) or sulforaphane (SFN; an Nrf2 activator) for 6 h in AML12 cells (data not shown). Immunostaining also showed that treatment with 100 μg/mL BLE or SFN for 6 h induced the nuclear translocation (activation) of Nrf2. In contrast, Nrf2 remained in the cytosol in the untreated control cells (Figure 4), indicating that BLE activated Nrf2 in AML12 cells. These results suggest that Nrf2-mediated the upregulation of antioxidant genes and that subsequent increase in cellular antioxidant activities is one of the mechanisms underlying the protective effect of BLE against ROS-induced cytotoxicity in AML12 cells.

### 3.3. Protective Effects of BLE against CCl_4_-Induced Acute Hepatotoxicity in BALB/c Mice

CCl_4_ has been widely used to induce acute and chronic liver injury in vivo, and injury caused by CCl_4_ is characterized mainly by excessive production of ROS, which eventually leads to hepatocellular damage [18]. Here, we examined whether BLE (100 and 400 mg/kg) can attenuate CCl_4_-induced acute hepatotoxicity in BALB/c mice. First, we observed that the weight gain and food intake in the BLE-treated groups significantly decreased (Table S2). Intraperitoneal injection of CCl_4_ led to a significant increase in the activity of serum alanine transaminase (ALT) and aspartate transaminase (AST). Pretreatment with 400 mg/kg BLE for 2 weeks significantly reduced serum levels of ALT, but not AST, and 100 mg/kg BLE did not show any significant changes in serum ALT and AST levels (Table 2). We also examined the effect of BLE on the levels of serum lipid profiles in CCl_4_-treated mice, and the result showed that only triglyceride (TG) levels were significantly increased by CCl_4_ treatment, which was significantly reduced by BLE (Table 2).

**Figure 5:**
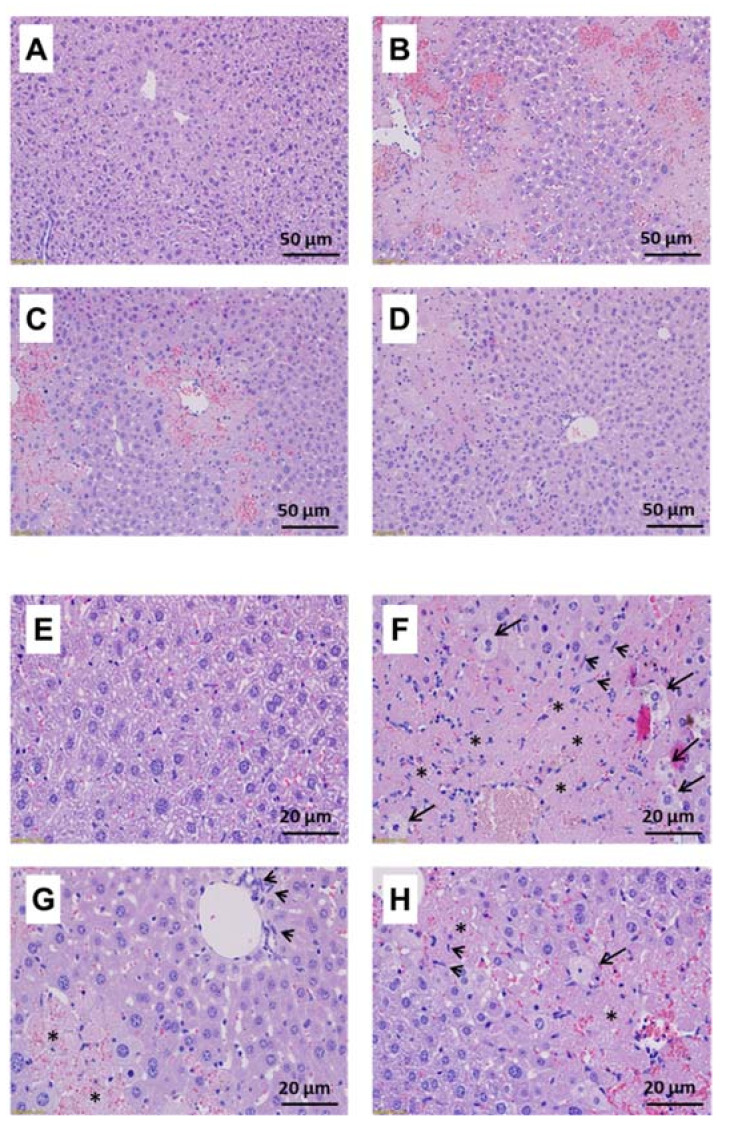
Effect of BLE on liver histology in CCl_4_-treated BALB/c mice. (**A**,**E**) Normal; (**B**,**F**) CCl_4_; (**C**,**G**) BLE100; (**D**,**H**) BLE400. Histologic sections were made from liver tissues and stained with hematoxylin and eosin. Representative images were collected at high magnification ((**A**–**D**) ×200; (**E**–**H**) ×400). The long arrow and the asterisk indicate hepatic injury (ballooning degeneration) and necrosis, respectively, and the short arrow indicates inflammatory cell infiltration.

To further assess the protective effects of BLE on CCl_4_-induced hepatotoxicity, histopathological examination of the liver tissues was performed. Liver weight did not vary significantly between the groups, and treatment with CCl_4_ and/or BLE did not cause hepatic fatty changes (data not shown). The CCl_4_-treated group showed a significant increase in the degree of injury as well as the levels of necrotic and inflammatory cell infiltration compared with the normal group. However, unlike changes in serum ALT and TG levels, the BLE-treated groups showed no significant changes in the levels of hepatic injury, necrosis, and inflammatory cell infiltration (Figure 5 and Table S3). However, the degree of injury was slightly lower in the BLE-treated groups than in the CCl_4_ group, but the difference was not statistically significant. These differences were probably due to severe hepatic damage and high animal-to-animal variation caused by the high dose of CCl_4_ [19], and therefore, it may be necessary to retest the hepatoprotective effects of BLE in a milder liver injury model.

**Figure 6:**
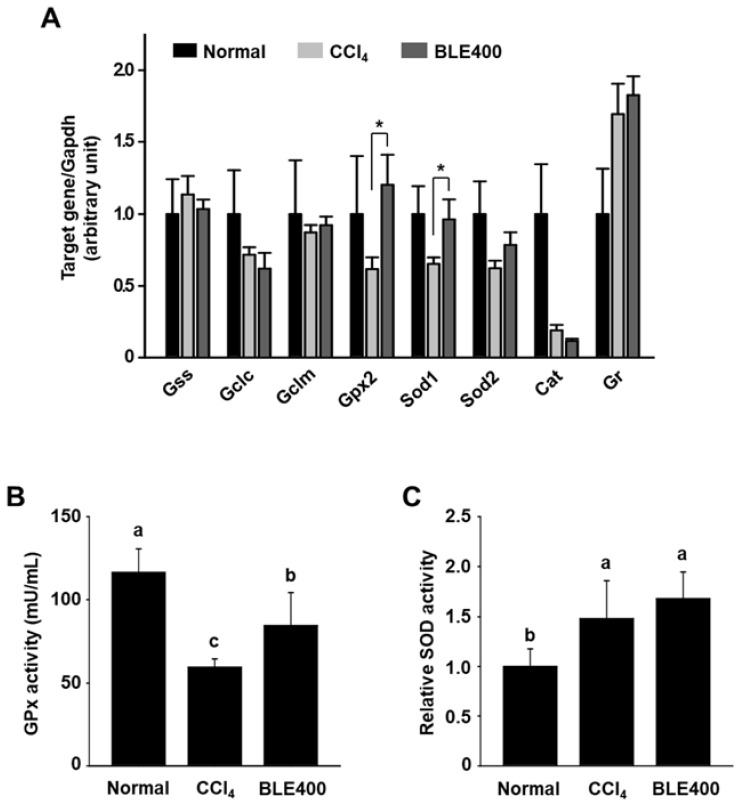
Effects of BLE on mRNA expression and activity of cellular antioxidant enzymes in liver tissues of CCl_4_-treated BALB/c mice. Liver homogenates were prepared as described in the Section 2. mRNA expression levels were determined by quantitative real-time PCR (**A**). Gapdh was used as an internal control. Enzyme activities in liver homogenates were measured (**B**,**C**). All results are expressed as mean ± SEM (Normal, *n* = 4; CCl_4_, *n* = 8; BLE400, *n* = 7). * *p* < 0.05 vs. the CCl_4_ group. Different letters are significantly different among groups, according to ANOVA with Duncan’s multiple range test (*p* < 0.05).

To study the in vivo mechanisms of action of BLE (400 mg/kg), we examined the mRNA expression levels of antioxidant genes in liver tissue. As shown in Figure 6A, only the gene expression of *Gpx2* and *Sod1* showed significant differences after treatment with BLE (400 mg/kg), which was somewhat different from the in vitro results. The mRNA expression of *Gpx2* and *Sod1* was dramatically decreased in the CCl_4_ group compared with the normal group, but in the BLE400 group, the expression levels of these two genes increased significantly and returned to the normal levels. The enzyme activity of Gpx2 was also significantly increased in the BLE400 group, compared with that in the CCl_4_ group (Figure 6B). Moreover, the activity of Sod1 showed a different pattern from the mRNA expression, and there was no change in activity following BLE treatment (Figure 6C). These results suggest that the protective effect of BLE against CCl_4_-induced acute hepatotoxicity in BALB/c mice may have been mediated by the increased activity of Gpx2 induced by the treatment with 400 mg/kg BLE, a mechanism different from that observed in H_2_O_2_-treated AML12 cells.

A recent study showed that red lentil extract (200 mg/kg) significantly decreased serum level of AST, ALT, and alkaline phosphatase and the activity of hepatic antioxidant enzymes such as SOD and catalase in sodium arsenite-treated Wistar rats, but the extract at 100 mg/kg did not restore all these serum markers and antioxidant enzymes [20]. Although there is a difference in the animal models and the hepatotoxic agents, these results are somewhat similar to our data, suggesting that the lentil extract can exhibit a more significant hepatoprotective effect at a dose of 200 mg/kg or more in animal models.

Reportedly, polyphenol-rich plants and polyphenolic compounds, such as flavonoids, carotenoids, and phenolic acid, exhibit protective effects against oxidative stress-induced hepatotoxicity by either the direct scavenging of ROS or the modulation of endogenous antioxidant defense system [21,22,23]. More recently, a study was conducted to elucidate the profile of phytochemical constituents in 20 different lentil cultivars and their antioxidant activities, and the results revealed that the content of total tocopherols and total carotenoids were 37–64 μg/g dry weight (DW) and 5.3–28.1 μg/g DW, respectively [24]. The results also demonstrated that the combination of tocopherols and carotenoids showed good correlation with 2,2-diphenyl-1-picrylhydrazyl radical scavenging activity (*r* = 0.6688). However, the phytochemical constituents and biological activities of beluga lentil have not been well studied. In our previous study, we demonstrated that the total phenolic and flavonoid contents of BLE (Table 1) did not differ significantly from those of other lentil cultivars such as red, green, and French lentils [6]. We also demonstrated that no significant difference was found in the protective effects of the four extracts against H_2_O_2_- and alcohol-induced cytotoxicity in AML12 cells (data not shown). Taken together, these results suggest that the phytochemical composition of beluga lentils is likely to be similar to that of other lentils. However, considering the difference in ROS scavenging activities by cultivar [6], it is also expected that there might be some differences in chemical composition, such as pigments that make up seed coats including anthocyanins [25].

## 4. Conclusions

In summary, this study demonstrates that BLE exerted protective effects against oxidative stress-induced cytotoxicity in AML12 cells by increasing cellular antioxidant capacity. In addition, BLE partially protected against CCl_4_-induced hepatotoxicity in a mouse model. This is the first study to demonstrate the hepatoprotective effects of beluga lentil and its underlying mechanisms, and the results of this study suggest that beluga lentil represents a potential source of natural hepatoprotective agents.

## Figures and Tables

**Table 1:** Content of the total polyphenols and flavonoids in BLE.

	Total Polyphenols (mg GAE ^(1)^/g Extract)	Total Flavonoids (mg QE ^(2)^/g Extract)
BLE	32.09 ± 0.37	19.32 ± 0.08

^(1)^ Gallic acid equivalent. ^(2)^ Quercetin equivalent. All results are expressed as means ± SD.

**Table 2:** Effects of BLE on serum levels of ALT, AST, and lipids in CCl_4_-treated BALB/c mice.

	Normal	CCl_4_	BLE100	BLE400
ALT (U/L)	73.15 ± 30.80 ^c^	9472.38 ± 2755.53 ^a^	10,420.50 ± 5724.78 ^a^	4860.90 ± 2041.11 ^b^
AST (U/L)	493.63 ± 334.75 ^b^	5158.45 ± 2333.14 ^a^	6829.04 ± 2995.22 ^a^	6010.76 ± 1140.44 ^a^
T-Chol (mg/dL)	141.50 ± 19.07 ^ns^	130.50 ± 20.13	132.43 ± 42.26	116.43 ± 20.28
TG (mg/dL)	169.50 ± 51.99 ^ab^	245.63 ± 81.19 ^a^	145.29 ± 81.40 ^b^	148.43 ± 46.97 ^b^
HDL-C (mg/dL)	105.73 ± 14.15 ^ns^	89.73 ± 12.15	99.43 ± 26.66	76.39 ± 30.46
LDL-C (mg/dL)	8.85 ± 1.78 ^ns^	11.05 ± 4.04	10.36 ± 3.52	17.93 ± 15.28

ns: not significant. All results are expressed as means ± SD (Normal, *n* = 4; CCl_4_, *n* = 8; BLE100 and BLE400, *n* = 7). Different letters are significantly different among groups, according to ANOVA with Duncan’s multiple range test (*p* < 0.05).

## Supplementary Materials

The following supporting information can be downloaded online, Table S1: Sequence of the primers used for qPCR; Table S2: Effects of BLE on body weight and food intake in BALB/c mice; Table S3: Effects of BLE on histopathological changes in liver tissues of CCl_4_-treated BALB/c mice.

## Sample Availability

Samples of the extract are available from the corresponding author.

## References

[1] Li S., Tan H.-Y., Wang N., Zhang Z.-J., Lao L., Wong C.-W., Feng Y. (2015). The Role of Oxidative Stress and Antioxidants in Liver Diseases. Int. J. Mol. Sci..

[2] Adewusi E.A., Afolayan A. (2010). A review of natural products with hepatoprotective activity. J. Med. Plants Res..

[3] Li Y., Hu D., Qi J., Cui S., Chen W. (2020). Lysosomal reacidification ameliorates vinyl carbamate-induced toxicity and disruption on lyso-somal pH. J. Agric. Food Chem..

[4] Lim J.-Y., Yun D.-H., Lee J.-H., Kwon Y.-B., Lee Y.-M., Lee D.-H., Kim D.-K. (2021). Extract of *Triticum aestivum* Sprouts Suppresses Acetaminophen-Induced Hepatotoxicity in Mice by Inhibiting Oxidative Stress. Molecules.

[5] Liu J., Li D., Zhang T., Tong Q., Ye R.D., Lin L. (2017). SIRT3 protects hepatocytes from oxidative injury by enhancing ROS scavenging and mito-chondrial integrity. Cell Death Dis..

[6] Lee S.-H. (2016). Polyphenol Contents and Antioxidant Activities of Lentil Extracts from Different Cultivars. J. Korean Soc. Food Sci. Nutr..

[7] Yang L., Wei J., Sheng F., Li P. (2019). Attenuation of Palmitic Acid–Induced Lipotoxicity by Chlorogenic Acid through Activation of SIRT1 in Hepatocytes. Mol. Nutr. Food Res..

[8] Köroğlu E., Canbakan B., Atay K., Hatemi I., Tuncer M., Dobrucalı A., Sonsuz A., Gültepe I., Şentürk H. (2021). Role of oxidative stress in the pathogenesis of non-alcoholic fatty liver dis-ease: Implications for prevention and therapy. Antioxidants.

[9] Ganesan K., Xu B. (2017). Polyphenol-Rich Lentils and Their Health Promoting Effects. Int. J. Mol. Sci..

[10] Li R., Wang Y., Zhao E., Wu K., Li W., Shi L., Wang D., Xie G., Yin Y., Deng M. (2016). Maresin 1, a Proresolving Lipid Mediator, Mitigates Carbon Tetrachloride-Induced Liver Injury in Mice. Oxid. Med. Cell. Longev..

[11] Yan C., Sun W., Wang X., Long J., Liu X., Feng Z., Liu J. (2016). Punicalagin attenuates palmitate-induced lipotoxicity in HepG2 cells by activating the Keap1-Nrf2 antioxidant defense system. Mol. Nutr. Food Res..

[12] López A., El-Naggar T., Dueñas M., Ortega T., Estrella I., Hernández T., Carretero M.E. (2017). Influence of processing in the phenolic composition and health-promoting properties of lintils (*Lens culinaris* L.). J. Food Process. Preserv..

[13] Yao F.-R., Sun C., Chang S.K.C. (2010). Morton Lentil Extract Attenuated Angiotensin II-Induced Cardiomyocyte Hypertrophy via Inhibition of Intracellular Reactive Oxygen Species Levels in Vitro. J. Agric. Food Chem..

[14] Xu B., Chang S.K.C. (2010). Phenolic Substance Characterization and Chemical and Cell-Based Antioxidant Activities of 11 Lentils Grown in the Northern United States. J. Agric. Food Chem..

[15] Kang H.W. (2015). Antioxidant activity of ethanol and water extracts from lentil (*Lens culinaris*). J. Food Nutr. Res..

[16] Nguyen T., Nioi P., Pickett C.B. (2009). The Nrf2-antioxidant response element signaling pathway and its activation by oxidative stress. J. Biol. Chem..

[17] Suzuki T., Yamamoto M. (2015). Molecular basis of the Keap1–Nrf2 system. Free. Radic. Biol. Med..

[18] Scholten D., Trebicka J., Liedtke C., Weiskirchen R. (2015). The carbon tetrachloride model in mice. Lab. Anim..

[19] Apte U. (2015). Liver Regeneration: Basic Mechanisms, Relevant Models and Clinical Applications.

[20] Kalantari H., Houshmand G., Hasanvand A., Kalantar M., Goudarzi M., Haghighian H.K. (2017). Ameliorative Effects of Red Lentil Extract on Sodium Arsenite-induced Oxidative Stress in Rats. Jundishapur J. Nat. Pharm. Prod..

[21] Abdel-Salam N.A., Ghazy N.M., Sallam S.M., Radwan M., Wanas A., ElSohly M.A., El-Demellawy M.A., Abdel-Rahman N.M., Piacente S., Shenouda M.L. (2017). Flavonoids of *Alcea rosea* L. and their immune stimulant, antioxidant and cytotoxic activities on hepatocellular carcinoma HepG-2 cell line. Nat. Prod. Res..

[22] Binu P., Gifty K., Vineetha R.C., Abhilash S., Arathi P., Nair R.H. (2018). Eugenol, a plant-derived phenolic nutraceutical, protects thiol (SH) group in myo-cardium from ROS-mediated oxidation under chemotherapeutic stress induced by arsenic trioxide—A in vivo model study. Drug Chem. Toxicol..

[23] Shahwar D., Bhat T.M., Ansari M.Y.K., Chaudhary S., Aslam R. (2017). Retracted Article: Health functional compounds of lentil (Lens culinaris Medik): A review. Int. J. Food Prop..

[24] Zhang B., Deng Z., Ramdath D.D., Tang Y., Chen P.X., Liu R., Tsao R. (2015). Phenolic profiles of 20 Canadian lentil cultivars and their contribution to antiox-idant activity and inhibitory effects on α-glucosidase and pancreatic lipase. Food Chem..

[25] Takeoka G.R., Dao L.T., Tamura H., Harden L.A. (2005). Delphinidin 3-O-(2-O-β-d-Glucopyranosyl-α-l-arabinopyranoside): A Novel Anthocyanin Identified in Beluga Black Lentils. J. Agric. Food Chem..

