# Supplementary material for: In Vitro and In Vivo Protective Effects of Lentil (Lens culinaris) Extract against Oxidative Stress-Induced Hepatotoxicity
Source: Molecules. 2021 Dec 23;27(1):59. doi: 10.3390/molecules27010059 (PMC8746911; doi:10.3390/molecules27010059)
Supplement: Supplementary file 1 [file molecules-27-00059-s001.zip › molecules-1462563-supplementary.pdf]

Supporting Information

Materials and methods

3 Tables

References

Materials and methods

1. Total polyphenol content

Total polyphenol content of BLE was measured using the modified method of Folin-Denis [23]. BLE (0.1 mL) was mixed with 0.1 mL of the Folin-Ciocalteu reagent (0.5 X), 1 mL of 10% (w/v) sodium carbonate, and 8.8mL of distilled water. After 1 h, the absorbance at 700 nm was measured with a UV/visible spectrophotometer (UVIKON 922, Germany). Gallic acid (10–100 μg/mL; R^2 = 0.9986) was used as the standard, and the results were expressed in mg/g gallic acid equivalents (GAE).

2. Total flavonoid content

Total flavonoid content was determined using the modified method of Nieva Moreno [1]. BLE (0.5 mL) was mixed with 0.1 mL of 10% (w/v) ethanolic solution of aluminum nitrate, 0.1 mL of 1 M potassium acetate and 4.3 mL of distilled water. After 40 min, the absorbance at 415 nm was measured using a UV/visible spectrophotometer (UVIKON 922, Germany). Quercetin (12.5 –400 μg/mL; R^2 = 0.9987) was used as the standard, and the results were expressed in mg/g quercetin equivalents (QE).

3. Cell viability assay

Cell viability was determined using the 3-(4,5-dimethylthiazol-2-yl)-2,5-diphenyltetrazolium bromide assay. Briefly, AML12 cells were plated in 96-well culture plates at a density of 1 × 10^4 cells/well in culture medium and allowed to attach for 24 h. The medium was then replaced with 200 μL of new medium containing various concentrations of BLE at 37 °C for 24 h. After incubation, the cells were washed and

treated with or without H_2O_2 (7 mM) for 1 h, and 10 μL of MTT stock solution (5 mg/mL in PBS) was added to each well and incubated for another 3 h. The media were then carefully removed, and insoluble purple formazan crystals were dissolved by adding 100 μL of DMSO, and the absorbance was measured at 540 nm.

4. Preparation of FFA-conjugated bovine serum albumin (BSA)

FFA stock solutions were prepared by conjugating free fatty acids with BSA. Briefly, sodium palmitate and sodium oleate were dissolved at a concentration of 250 mM. This FFA stock solution was then added to a pre-warmed 11% w/v BSA solution (37 °C) to achieve a FFA concentration of 25 mM, and this solution was allowed to incubate in a water bath for an additional 10 min.

5. Measurement of intracellular ROS levels

The levels of intracellular ROS were measured by flow cytometry using the peroxide-sensitive fluorescent probe 2'7'-dichlorofluorescin diacetate (DCF-DA) as previously described [2]. Briefly, AML12 cells were treated with BLE for 24 h and the medium was replaced with new medium containing H_2O_2 (7 mM). After 1 h incubation with H_2O_2, the cells were washed with PBS and incubated with 25 μM DCF-DA for 30 min at 37 °C. The cells were then washed twice with PBS and detached by treatment with trypsin-EDTA. The detached cells were collected and resuspended in PBS, and the fluorescence intensity of the cells was measured using flow cytometry (BD FACSVerse, BD-Biosciences, San Jose, CA).

6. Quantitative real-time polymerase chain reaction (PCR) and western blot analyses

Quantitative real-time PCR and western blot analyses were performed as previously described [3]. Table S1 shows the primer sequences. The intensity of the protein bands was quantitated using ImageJ.

7. Measurement of the serum levels of AST, ALT, and lipids

The serum levels of AST, ALT, and lipids (total cholesterol, triglyceride, HDL-cholesterol, and LDL-cholesterol) were measured by DooYeol Biotech (Seoul, Korea).

8. Histopathological evaluation of liver injury

For histological liver damage score, an expert liver pathologist blindly reviewed all samples. A detailed histological analysis was prospectively performed as follows: (1) injury: presence of balloon cells, Mallory bodies, feathery degeneration, (2) necrosis, apoptosis (apoptotic body, acidophil body, or councilman body); (3) fatty change: steatosis; and (4) inflammatory cell infiltration: presence of macrophages, T lymphocytes, neutrophils, and dendritic cells.

9. Determination of GSH content and antioxidant enzyme activity

To determine GSH content and enzyme activities, cell lysates and liver homogenates were prepared in a reporter lysis buffer (Promega, St. Wisconsin, Madison) and ice-cold 0.1 M potassium phosphate buffer (pH 7.5), respectively. The protein content was determined using a BCA assay kit (Sigma-Aldrich Chemical, St. Louis, MO) according to the manufacturer's instructions. GSH content was determined by the

enzymatic method as previously described [4]. GSH content was measured by an increase in absorbance at 412 nm for 5 min in a final 1 mL reaction mixture containing 0.1 M sodium phosphate buffer (pH 7.6), 5 μM EDTA, 0.6 mM 5,5-dithiobis (2-benzoic acid), 0.2 mM NADPH, 1 unit/mL GR, and 10 μL lysate. The level of malondialdehyde was measured as previously described [4]. GR activity and GPx activity were measured using a commercially available GR assay kit (Cayman Chemical Co., Ann Arbor, MI) and a GPx assay kit (Abcam, Cambridge, MA), respectively. One unit of GR represents 1 μmol of NADPH consumed/min, and its specific activity was expressed as nmol NADPH/min/mg protein. Catalase and SOD activities were measured as previously described [4].

Table S1. Sequence of the primers used for qPCR

Target gene		Primer sequence
Gapdh	Sense	5’-GTATGACTCCACTCACGGCA-3’
	Antisense	5’-GGTCTCGCTCCTGGAAGATG-3’
Gss	Sense	5’-GAAGCAGCTCGAAGAACTGG-3’
	Antisense	5’-AGCACTGGGTACTGGTGAGG-3’
Gclc	Sense	5’-CTGCACATCTACCACGCAGT-3’
	Antisense	5’-GTCTCAAGAACATCGCCTCC-3’
Gclm	Sense	5’-CCAAAACATCTGGAAACTCCC-3’
	Antisense	5’-CGGGAACCTGCTCAACTG-3’
Sod1	Sense	5’-ACCATCCACTTCGAGCAGAA-3’
	Antisense	5’-AAAATGAGGTCCTGCACTGG-3’
Sod2	Sense	5’-AACTCAGGTCGCTCTTCAGC-3’
	Antisense	5’-GCTTGATAGCCTCCAGCAAC-3’
Gpx2	Sense	5’-AGGTCGGACATACTTGAGGC-3’
	Antisense	5’-GGTAGTTCTCGGCTTCCCTT-3’
Cat	Sense	5’-CCCGCGGTCATGATATTAAGT-3’
	Antisense	5’-GATGAAGCAGTGGAAGGAGC-3’
Gr	Sense	5’-ATCGTGCATGAATTCCGAGT-3’
	Antisense	5’-GGTGGTGGAGAGTCACAAGC-3’

Gapdh; Glyceraldehyde 3-phosphate dehydrogenase, Gss; glutathione synthetase, Gclc; glutamate-cysteine ligase catalytic subunit, Gclm; glutamate-cysteine ligase modifier subunit, Sod1; superoxide dismutase 1 (soluble), Sod2; superoxide dismutase 2 (mitochondrial), Gpx2; glutathione peroxidase 2, Cat; catalase, Gr; glutathione reductase.

Table S2. Effects of BLE on body weight and food intake in BALB/c mice

	Normal	CCl_4	BLE100	BLE400
Body weight gain (g/week)	2.51±0.53^{a}	2.19±0.10^{ab}	1.46±0.44^{bc}	0.57±0.77^{c}
Food intake (g/week)	37.38±1.94^{a}	34.09±4.42^{ab}	29.79±4.87^{b}	28.12±1.62^{b}

All results are expressed as means ± SD (Normal, n=4; CCl_4, n=8; BE100 and BE400, n=7). Different letters are significantly different among groups, according to ANOVA with Duncan's multiple range test ($P < 0.05$).

Table S3. Effects of BLE on histopathological changes in liver tissues of CCl_4-treated BALB/c mice

	Normal	CCl_4	BLE100	BLE400
Injury (%)	ND*	9.69±11.13ns**	2.50±0.00	6.43±6.51
Necrosis (%)	ND	30.00±11.95ns	34.29±15.12	32.86±14.96
Inflammatory cell infiltration (%)	ND	22.50±10.35ns	18.57±12.15	20.00±10.00

*ND: not detected.

**ns: not significant.

All results are expressed as means ± SD (Normal, n=4; CCl_4, n=8; BE100 and BE400, n=7).

References

1. V.L. Singleton, Naturally occurring food toxicants: phenolic substances of plant origin common in foods. Adv. Food Res. 27 (1981) 149-242.
2. M.I. Mieva Moreno, M.I. Isla, A.R. Sampietro, M.A. Vattuone, Comparision of the free radical-scavenging activity of propolis from several regions of Argentina. J. Ethnopharmacol. 71 (2000) 109-114.
3. H.S. Lee, S. Safe, S.O. Lee, Inactivation of the orphan nuclear receptor NR4A1 contributes to apoptosis induction by fangchinoline in pancreatic cancer cells. Toxicol. Appl. Pharmacol. 332 (2017) 32-39.
4. E.Y. Hwang, M.H. Yu, Y.S. Jung, et al., Defatted safflower seed extract inhibits adipogenesis in 3T3-L1 preadipocytes and improves lipid profiles in C57BL/6J ob/ob mice fed a high-fat diet. Nutr. Res. 36 (2016) 995-1003.
5. S.O. Lee, H.W. Lee, I.S. Lee, et al., The pharmacological potential of Sorbus commixta cortex on blood alcohol concentration and hepatic lipid peroxidation in acute alcohol-treated rats. J. Pharm. Pharmacol. 58 (2006) 685-693.
